# The Evolution of Molecular Phenotypes Through Time in Kidney Transplant Recipients

**DOI:** 10.1097/TXD.0000000000001919

**Published:** 2026-03-23

**Authors:** Brigitte Baragar, Benjamin A. Adam, Michael Mengel, Robert Balshaw, Ian W. Gibson, Julie Ho, James Shaw, Martin Karpinski, Aaron Trachtenberg, Denise Pochinco, Aviva Goldberg, Patricia Birk, Maury Pinsk, David Rush, Peter W. Nickerson, Chris Wiebe

**Affiliations:** 1 Department of Medicine, University of Manitoba, MB, Canada.; 2 Department of Laboratory Medicine and Pathology, University of Alberta, AB, Canada.; 3 George and Fay Yee Centre for Healthcare Innovation, University of Manitoba, MB, Canada.; 4 Shared Health Services, MB, Canada.; 5 Department of Pathology, University of Manitoba, MB, Canada.; 6 Department of Immunology, University of Manitoba, MB, Canada.; 7 Department of Pediatrics and Child Health, University of Manitoba, MB, Canada.

## Abstract

**Background.:**

The correlation between histologic lesions and allograft gene expression data is a subject of significant interest. Most analyses include a single for-cause biopsy from each recipient, precluding serial assessment of gene scores.

**Methods.:**

Using the nCounter system, we evaluated the Banff Human Organ Transplant gene panel in a unique cohort of 8 renal transplant recipients, each with 3 distinct histologic phenotypes (no rejection, T cell–mediated rejection [TCMR], and antibody-mediated rejection [AMR]), to explore the serial evolution of molecular phenotypes.

**Results.:**

No rejection biopsies showed large interindividual differences in TCMR and AMR gene scores. Compared with biopsies with No Rejection, TCMR gene scores were greater in those with TCMR (mean fold change 1.95 ± 0.87, *P* = 0.0174). Similarly, compared with biopsies with No rejection, gene scores for AMR (mean fold change 1.43 ± 0.24, *P* = 0.0002), donor-specific antibody selective transcripts (mean fold change 1.33 ± 0.22, *P* = 0.002), and endothelial cell–associated transcripts (mean fold change 1.17 ± 0.06, *P* = 0.04) were greater at the time of AMR.

**Conclusions.:**

In most cases, deviations in gene scores occurred in the expected direction as recipients transitioned through histologic phenotypes. The unexpected deviations in gene scores and the significant variability among individuals within histologic phenotypes warrant further investigation.

## INTRODUCTION

Timely diagnosis of T cell–mediated rejection (TCMR) and antibody-mediated rejection (AMR) is critical in kidney transplant care, as they remain major causes of allograft injury and failure.^1,2^ Currently, the gold standard for the diagnosis of allograft rejection is histology.^3^ However, histologic classification is subject to interobserver variability, sample bias, and ambiguity, limiting its diagnostic accuracy.^4,5^

Biopsy-based transcriptomics has emerged as a tool to complement histologic evaluation, providing molecular-based assessment of allograft tissue.^6-8^ The Molecular Microscope Diagnostic System (MMDx), developed by Halloran et al, has contributed novel insights to the molecular pathways involved in various rejection states.^8-11^ Multicentric prospective trials have explored the potential diagnostic application of the MMDx, with promising results for accurately diagnosing AMR and TCMR.^6,7,9,10,12^ Recent studies have demonstrated the potential of MMDx to predict the development of rejection in protocol biopsies and monitor antirejection treatment responses.^13,14^ The Bruker nCounter Analysis System (formerly by NanoString Technologies, Seattle, WA) is an alternative method of transcriptome analysis using predefined gene sets. It offers the advantage of reliably quantifying RNA expression in preexisting histologic samples or archived formalin-fixed paraffin-embedded (FFPE) tissue, allowing direct molecular-histologic correlation.^15,16^ To facilitate further study of this technique, the Molecular Diagnostics Working Group of the Banff Foundation for Allograft Pathology developed the Banff Human Organ Transplant (B-HOT) panel, a 770-gene set including markers of tissue damage, organ rejection, and immune response.^17^

Since the creation of the B-HOT panel, several investigators have applied molecular classifiers using the nCounter system to characterize the molecular phenotypes of histologic AMR and TCMR.^6,18-21^ Recently, Loupy et al^22^ developed a B-HOT-based predictive model, similar to the MMDx, in which automated diagnostic reports provide probabilistic scores supporting or rejecting a diagnosis of rejection. However, it remains unclear how gene expression changes over time within a given recipient, as most commonly, analyses using this system have included a single for-cause biopsy from each recipient. Furthermore, how such gene expression patterns can be used clinically for diagnostic and patient management purposes requires further study, as diagnostic thresholds for molecular AMR and TCMR have yet to be established or validated using the B-HOT panel.^23^

This case series used the B-HOT panel to evaluate a unique cohort of 8 renal transplant recipients, each of whom displayed all 3 distinct histologic phenotypes over time (no rejection, TCMR, and AMR) to explore the evolution of molecular phenotypes within the individual. Our objective was to investigate fluctuations in B-HOT gene scores as the histologic phenotypes change over time, and to assess whether these scores exhibit true dynamism or remain consistently elevated in recipients who encounter rejection episodes.

## MATERIALS AND METHODS

### Study Population

Written informed consent was not required for this retrospective analysis of archival biopsy material because the study posed minimal risk and did not involve identifiable patient information, and our Institutional Review Board waived the requirement for consent (H2023:020). Seventy-six adult and pediatric archived renal allograft biopsies collected in Manitoba between March 1999 and March 2021 were considered for inclusion in the case series. All recipients developed de novo donor-specific antibody (dnDSA) during the inclusion period for the study and had a variety of histologic phenotypes, including no rejection, TCMR alone, AMR alone, and mixed rejection (TCMR and AMR). dnDSA was detected using a solid-phase HLAs antibody assay. All dnDSAs had mean fluorescence intensity (MFI) ≥1000. Standard immunosuppression consisted of a calcineurin inhibitor, mycophenolate mofetil, and prednisone. Treatment of TCMR included optimizing tacrolimus (trough level 8 ± 2 ng/mL) and mycophenolate dose, a steroid bolus with taper, and, in cases of severe clinical TCMR, thymoglobulin (4–6 mg/kg). Treatment for acute clinical AMR included high-dose IVIG (2 g/kg), plasmapheresis, and steroids. Demographic, clinical, and histologic information was obtained from archived records.

### RNA Isolation and Gene Expression Analysis

RNA was isolated from archived FFPE renal allograft biopsy samples using the RNeasy FFPE Kit (Qiagen, Toronto, ON) at the University of Alberta. The concentration and purity of isolated RNA were determined using the NanoDrop 2000c spectrophotometer (Thermo Fisher Scientific, Waltham, MA). Gene expression was quantified using the Bruker nCounter Analysis System (formerly by NanoString Technologies, Seattle, WA) and the B-HOT gene panel.^17^

Molecular phenotype was analyzed by calculating previously published gene scores and comparing them to histologic and/or clinical phenotypes. Gene scores were determined through the calculation of the geometric means of the quantified select transcripts. Gene scores included TCMR,^19^ AMR, donor-specific antibody selective transcripts (DSASTs), and endothelial cell–associated transcripts (ENDATs).^17^ The previously published clinical relevance of these gene scores can be found in **Table S1** (**SDC,** https://links.lww.com/TXD/A837). Individual genes comprising each of the gene scores are shown in **Table S2** (**SDC,** https://links.lww.com/TXD/A837.

### Statistical Analysis

Gene scores were calculated as geometric means for each of the gene sets of interest (TCMR, AMR, etc; see **Tables S1 and S2, SDC,** https://links.lww.com/TXD/A837). The gene scores were then base-10 logarithmically transformed before calculating change scores and paired *t* tests for the significance of the within-patient changes; change scores were compared between the AMR and mixed AMR groups using a 2-sample *t* test. Each test was performed at the 0.05 level of significance without correction for multiple inference. Means and confidence intervals (CIs) for the log-10 transformed scores were then back-transformed using base-10 exponentiation to yield geometric means and fold changes on the original scale. Analyses were performed using JMP Pro version 18.2.0.

## RESULTS

### Total Study Population

**FIGURE 1.**
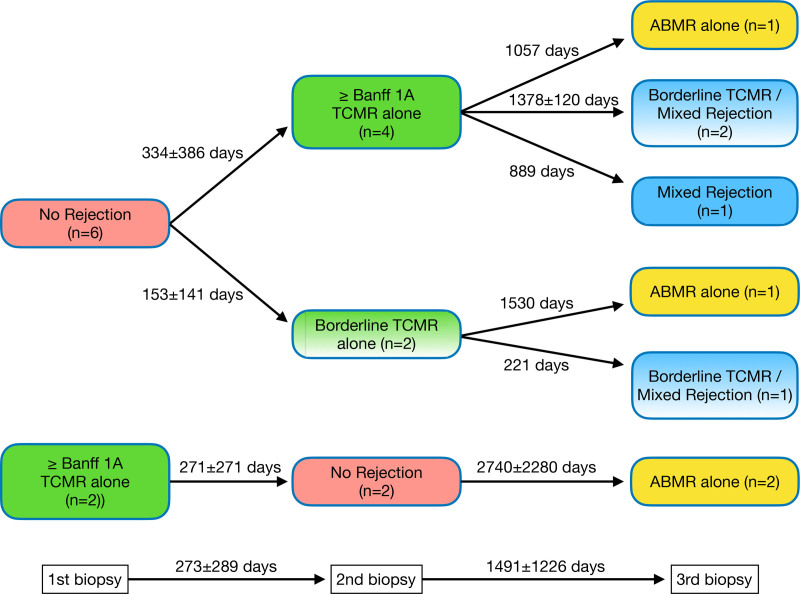
Histologic phenotype chronological order in recipients (n = 8) with 3 distinct serial biopsies. Time difference between biopsies represented in days. The mean time between the first and second, and the second and third biopsies is shown at the bottom of the figure.

Of the 76 adult and pediatric archived renal allograft biopsies considered for inclusion, 8 recipients each had 3 distinct histologic phenotypes at different time points (no rejection, TCMR, and AMR or mixed rejection) and were included in our case series. The mean time between the first and second biopsy was 273 ± 289 d, whereas the mean time between the second and third biopsy was 1491 ± 1226 d. More detailed biopsy timelines, with biopsies in chronological order, are presented in Figure 1. The average age of the transplant recipients was 17 y (range, 9–31). Relevant demographic and clinical characteristics are presented in Table 1. DSA specificities can be found in **Table S3** (**SDC,** https://links.lww.com/TXD/A837). All recipients had a biopsy showing histologic no rejection (n = 8) and TCMR alone (n = 8). In addition, 4 (50%) had a third biopsy showing AMR alone, and 4 (50%) had a biopsy showing mixed rejection. The Banff TCMR grade was borderline in 3 of 4 biopsies with mixed rejection and 2 of 8 biopsies with TCMR alone. The order of histologic phenotypes is shown in Figure 1. Histologic lesion scores are presented in Table 2.

**TABLE 1.** Demographic and clinical characteristics of recipients (n = 8) with serial biopsies

Characteristics	N = 8
Male donor	50%
Donor Age (y)	39 ± 11
Deceased donor	38%
Previous transplant	13%
Male recipient	63%
Recipient age, y	17 ± 7
White ethnicity	75%
Delayed graft function	13%
Induction immunosuppression	
Basiliximab	50%
Thymoglobulin	25%
None	25%
Noninduction immunosuppression	
Tacrolimus + mycophenolate + prednisone	100%
HLA-DR/DQ single-molecule eplet mismatch risk category	
Low	13%
Intermediate	13%
High	75%

**TABLE 2.** Histologic Banff lesion scores in serial biopsies (n = 24) among the 8 recipients

Banff histologic lesion score		No rejection (N = 8)	TCMR alone (N = 8)	Mixed rejection (N = 4)	AMR alone (N = 4)
Acute					
Glomerulitis	g	0 ± 0	0.1 ± 0.4	1.0 ± 1.4	0.8 ± 1.0
Interstitial inflammation	i	0 ± 0	2.1 ± 0.8	2.5 ± 1.0	0.8 ± 1.0
Tubulitis	t	0 ± 0	2.1 ± 0.6	1.5 ± 1.0	0.25 ± 0.5
Intimal arteritis	v	0 ± 0	0.1 ± 0.4	0 ± 0	0 ± 0
Peritubular capillarities	h	0 ± 0	2.2 ± 0.8	2.5 ± 0.6	1.5 ± 1.3
C4d positive		0%	0%	50%	75%
Chronic					
Glomerular basement membrane double contours	cg	0 ± 0	0 ± 0	1.0 ± 1.4	1.0 ± 1.4
Interstitial fibrosis	ci	0.7 ± 0.5	0.7 ± 0.5	2.0 ± 0.8	1.3 ± 0.5
Tubular atrophy	ct	0.9 ± 0.4	1.0 ± 0	2.5 ± 0.6	1.3 ± 0.5
Vascular fibrous intimal thickening	cv	0.7 ± 0.7	0.3 ± 0.5	1.0 ± 0.8	0.8 ± 0.5
Arteriolar hyalinosis	ah	0.3 ± 0.6	0 ± 0	NA	1.0 ± 1.4
Composite lesion scores					
i+t		0 ± 0	4.3 ± 1.4	4.0 ± 1.6	1.0 ± 0.8
g+ptc		0 ± 0	2.3 ± 1.0	3.5 ± 1.9	2.3 ± 1.3
g+i+t+v+ptc		0 ± 0	6.8 ± 1.0	7.5 ± 1.0	3.3 ± 1.7
ci+ct		1.6 ± 0.7	1.8 ± 0.5	4.5 ± 1.3	2.5 ± 1.0
cg+ci+ct+cv		2.3 ± 1.6	2.0 ± 0.8	6.5 ± 3.1	4.3 ± 1.3

AMR, antibody-mediated rejection; TCMR, T cell–mediated rejection.

### Molecular Gene Scores and Histologic Phenotype

**FIGURE 2.**
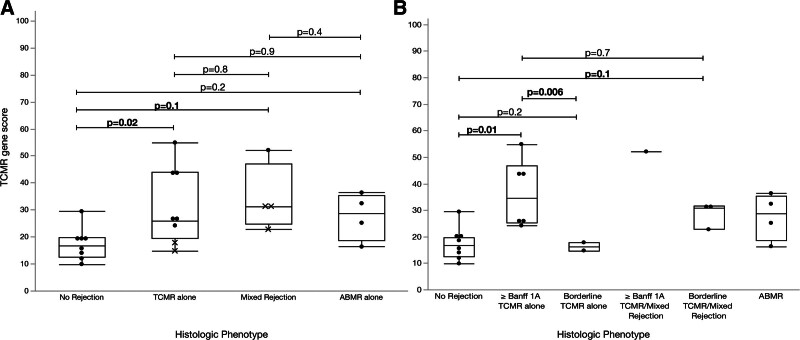
TCMR gene scores compared with histologic phenotype in recipients (n = 8) with serial biopsies. A, Box-plot displaying distribution with borderline TCMR represented by marker, X. B, Box-plot displaying distribution with borderline TCMR as a distinct category. TCMR, T cell–mediated rejection.

**FIGURE 3.**
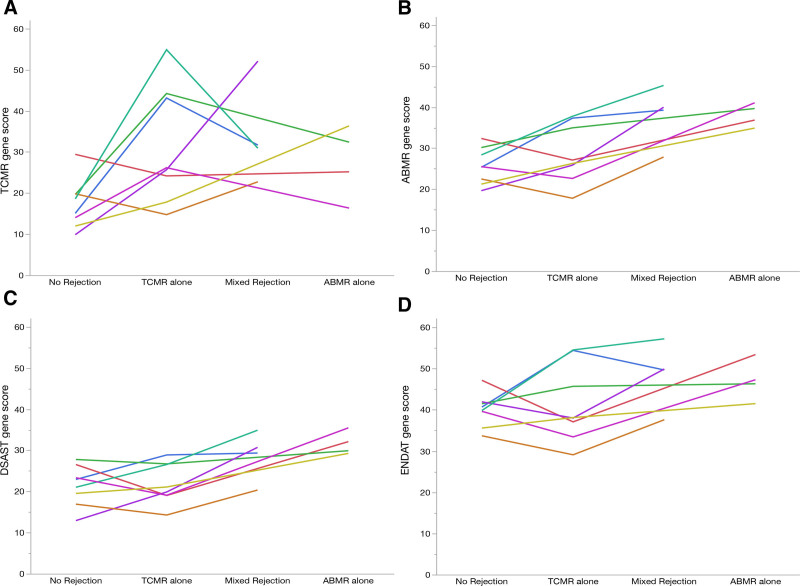
Gene score changes by histologic phenotype for each recipient (n = 8): TCMR gene score (A), AMR gene score (B), DSAST gene score (C), and ENDAT gene score (D). AMR, antibody-mediated rejection; DSAST, donor-specific antibody selective transcript; ENDAT, endothelial-associated transcript; TCMR, T cell–mediated rejection.

Compared with biopsies with no rejection, TCMR gene scores were greater in those with TCMR (n = 8; 17.3 versus 31.4; *P* = 0.02; Figure 2), a mean fold change of 1.74 (95% CI, 1.1-2.7; Figure 3). This correlation was stronger when borderline TCMR biopsies (n = 2) were excluded from the analysis (17.8 versus 36.4, *P* = 0.01; Figure 2), yielding a mean fold change of 2.05 (95% CI, 1.2-3.4). Compared with biopsies with borderline TCMR, the TCMR gene scores were greater in those with Banff ≥1A TCMR (16.3 versus 36.4, *P* = 0.006; Figure 2). There was no significant difference in TCMR gene scores when biopsies with borderline TCMR (n = 2) were compared with biopsies with No Rejection. Compared with biopsies with No Rejection, TCMR gene scores were nonsignificantly greater in those with mixed rejection (n = 4; 15.8 versus 34.4; *P* = 0.1; Figure 2), a mean fold change of 2.14 (95% CI, 0.8-6.0; Figure 3). Small numbers limited the ability to analyze the mixed rejection group without borderline TCMR histology. TCMR gene scores did not differ between biopsies with TCMR and those with AMR (Figure 2).

**FIGURE 4.**
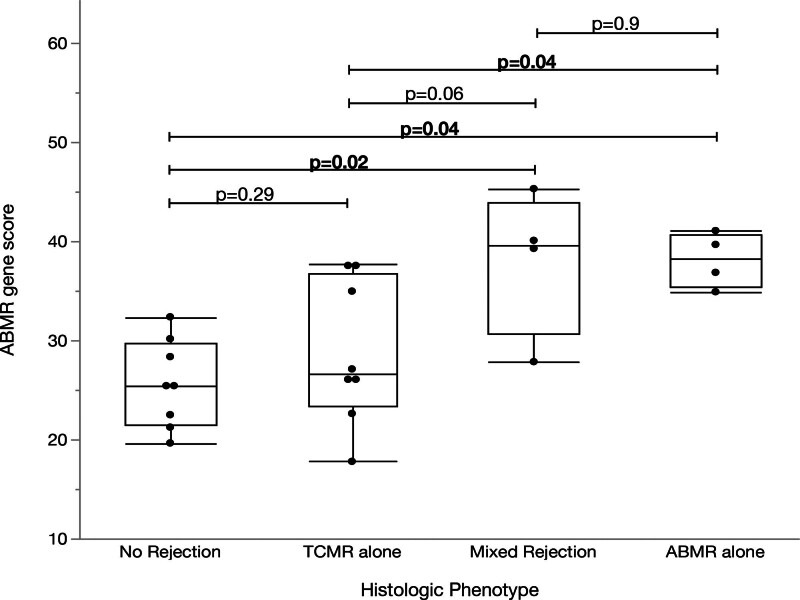
AMR gene scores compared with histologic phenotype in recipients (n = 8) with serial biopsies. AMR, antibody-mediated rejection.

Compared with biopsies with no rejection, AMR gene scores were greater in those with AMR (n = 4; 27.3 versus 38.1, *P* = 0.04; Figure 4), with a mean fold change of 1.39 (95% CI, 1.0-1.9; Figure 3). Compared with biopsies with no rejection, AMR gene scores were greater in those with mixed (n = 4, 24.0 versus 38.1; *P* = 0.02; Figure 4), with a mean fold change of 1.58 (95% CI, 1.1-2.2; Figure 3). Compared with biopsies with TCMR, AMR gene scores were also increased in AMR (n = 4; 27.7 versus 38.1; *P* = 0.04; Figure 4), with a mean fold change of 1.39 (95% CI, 1.0-1.9; Figure 3). There was a nonsignificant increase in AMR gene scores in mixed rejection compared with TCMR (n = 4; 29.7 versus 38.1; *P* = 0.06; Figure 4), with a mean fold change of 1.32 (95% CI, 0.97-1.8; Figure 3). dnDSAs included class I alone (n = 1), class II alone (n = 2), and class I and II (n = 5), with MFI ranging from 1779 to 12 341 (**Table S3, SDC,** https://links.lww.com/TXD/A837). There were no significant differences in the AMR or DSAT gene score by dnDSA Class, MFI, or dnDSA specificity.

**FIGURE 5.**
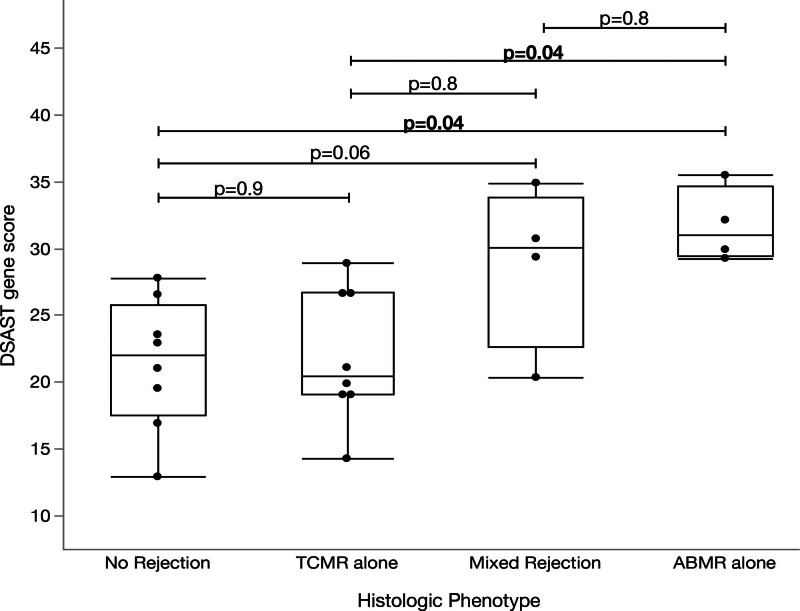
DSAST gene scores compared with histologic phenotype in recipients (n = 8) with serial biopsies. DSAST, donor-specific antibody selective transcript.

Compared with biopsies with no rejection, DSAST gene scores were greater in those with AMR (n = 4; 21.3 versus 31.7; *P* = 0.04; Figure 5), with a mean fold change of 1.32 (95% CI, 1.0-1.7; Figure 3). Compared with biopsies with no rejection, DSAST gene scores were nonsignificantly greater in those with mixed AMR (n = 4; 18.4 versus 28.8; *P* = 0.06; Figure 5), with a mean fold change of 1.57 (95% CI, 0.9-2.6; Figure 3). DSAST gene scores were also greater in AMR (n = 4; 31.7 versus 21.9; *P* = 0.04) compared with TCMR, with a mean fold change of 1.49 (95% CI, 1.0-2.1). There was a nonsignificant increase in DSAST gene scores in mixed AMR compared with TCMR (n = 4; 28.8 versus 21.9; *P* = 0.06; Figure 5), with a mean fold change of 1.31 (95% CI 0.9-1.7; Figure 3).

**FIGURE 6.**
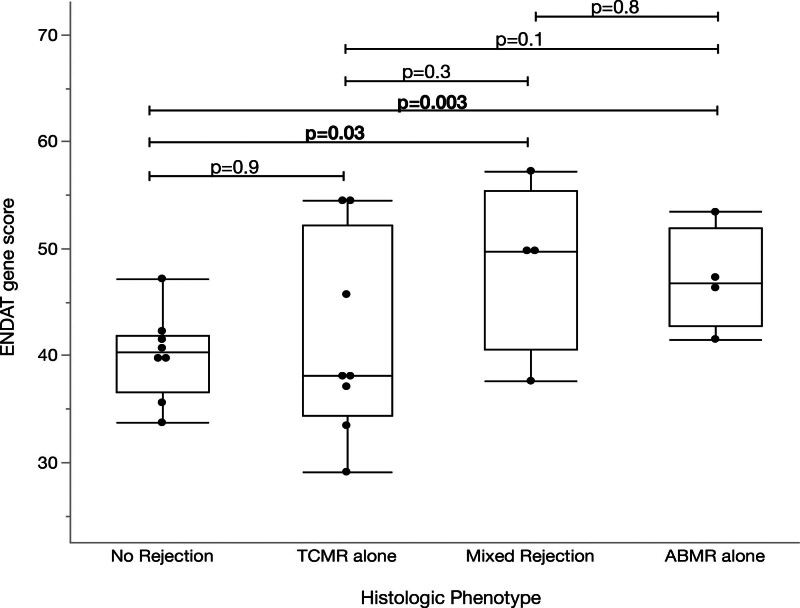
ENDAT gene scores compared with histologic phenotype in recipients (n = 8) with serial biopsies. ENDAT, endothelial-associated transcript.

Compared with biopsies with no rejection, ENDAT gene scores were greater in those with AMR (n = 4; 40.1 versus 47.1; *P* = 0.003; Figure 6), with a mean fold change of 1.15 (95% CI, 1.1-1.2; Figure 3). Compared with biopsies with no rejection, ENDAT gene scores were also greater with mixed AMR (n = 4; 39.0 versus 48.6; *P* = 0.03; Figure 6) with a mean fold change of 1.24 (95% CI, 1.0-1.5; Figure 3). ENDAT scores were not significantly changed when comparing TCMR with AMR or mixed AMR (Figure 6).

### Molecular Gene Scores Within the Individual

The molecular gene score deviated in the expected direction corresponding to the histologic phenotype, in 14 of 16 rejection events. AMR, DSAST, and ENDAT gene scores increased in all recipients (8/8) when histologic no rejection was compared with AMR. TCMR gene score increased in most (6/8) recipients when histologic no rejection was compared with TCMR (Figure 3).

### Interindividual Variation in Molecular Gene Scores

**FIGURE 7.**
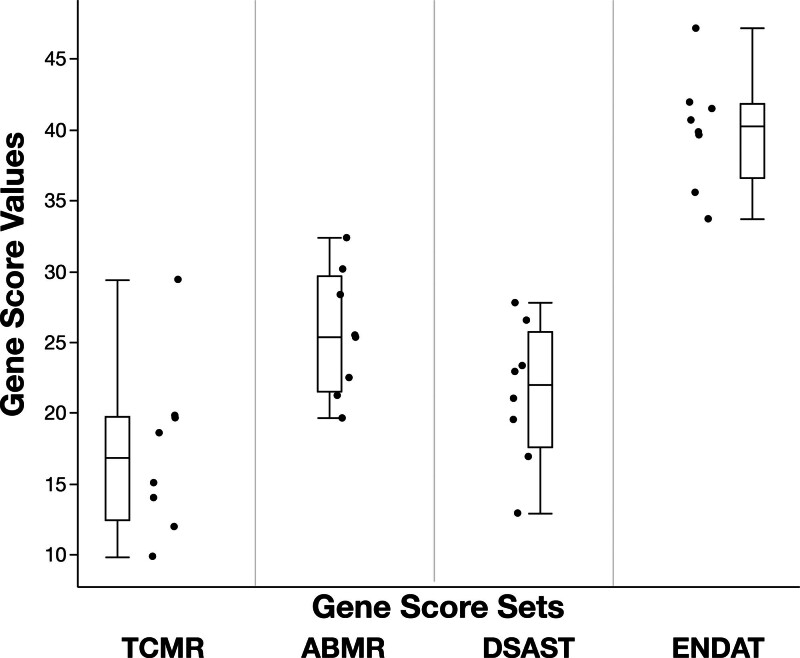
Distribution of TCMR and AMR gene scores among no rejection biopsies (n = 8). AMR, antibody-mediated rejection; TCMR, T cell–mediated rejection.

Differences in the molecular gene scores of no rejection biopsies (n = 8) existed despite their histologic similarity (Table 2). Ranges of the TCMR, AMR, DSAST, and ENDAT gene scores among the no rejection biopsies were 9.9–29.4, 19.6–32.4, 12.9–27.8, and 33.4–47.2, respectively (Figure 7), a 1.4-fold (ENDAT) to 2.9-fold (TCMR) difference. This variation could not be explained by the Banff g, i, t, v, and ptc scores (Table 2).

## DISCUSSION

This is the first study to evaluate molecular gene scores in serial biopsies from the same individuals using the Bruker nCounter Analysis System. Two recent studies have evaluated the molecular phenotypes of 2 consecutive biopsies, a baseline biopsy and a follow-up biopsy, among transplant recipients using the MMDx to characterize early molecular rejection signals.^13,14^ Our study evaluated 3 consecutive biopsies from 8 transplant recipients over time, strengthening the concept that molecular phenotypes evolve in parallel with the histologic diagnoses and highlighting that molecular phenotypes are not static over time.

Despite the small sample size, a significant correlation was observed between the TCMR gene score and histologic TCMR and mixed rejection, in keeping with previous literature.^19,24^ However, the correlation lost statistical significance when the comparison was restricted to borderline TCMR and no rejection biopsies, as borderline TCMR showed lower TCMR scores on average (Figure 2). This has been previously described in studies using the microarray technique, in which borderline TCMR often does not meet the diagnostic threshold for molecular TCMR.^25,26^ Rosales et al^20^ showed that aggregate first principal component (PC1) scores were statistically different between borderline TCMR and TCMR biopsies in a discovery study.^20^ However, as a discovery study, no attempts were made to generate thresholds applicable to other cohorts, and comparing PC1 of a diverse group of samples is not applicable to small sample sizes or individual biopsies. Although the small sample size in our study may have limited the ability to detect such differences, additional validation studies of borderline rejection are warranted, as this is an instance where molecular gene scores could provide the greatest clinical utility. Our results demonstrated that AMR, DSAST, and ENDAT gene scores could distinguish AMR or mixed rejection from no rejection, despite only 4 pairwise comparisons per score, consistent with previous literature.^19,27^ AMR and DSAST gene scores were also significantly increased in AMR versus TCMR; however, mixed AMR was not significantly different than TCMR.

Gene scores increased or decreased in parallel with histologic phenotypes in most recipients. For example, the AMR, DSAST, and ENDAT gene scores were higher in AMR than in no rejection in all recipients (8/8), and the TCMR gene scores were higher in TCMR than in no rejection in most cases (6/8). Two recipients had TCMR gene scores that were lower in the TCMR biopsy compared with the no rejection biopsy (Figure 3). The first recipient transitioned from a g0, i1, t0, v0, and ptc0 no rejection biopsy to a g0, i1, t1, v0, and ptc0 borderline TCMR biopsy with similar TCMR gene scores (19.8 versus 14.8, respectively). As stated previously, the TCMR gene score may be unable to discriminate between these subtle histologic differences (Figure 2B). The second recipient developed dnDSA 15 mo before the no rejection biopsy, with Banff acute scores of g1, i0, t0, v0, ptc0, and was C4d-negative. Notably, the AMR and TCMR scores from this biopsy were the highest among the subset of biopsies without rejection. A subsequent surveillance biopsy 1 y later, while function remained stable, showed mixed TCMR and AMR (g1, i2, t2, ptc0, and C4d-positive). Therefore, the gene scores in this case may indicate early, evolving mixed rejection that is below the Banff histologic thresholds and insufficient to cause clinical deterioration. This case may serve as an example of a situation in which molecular scores demonstrated a greater ability to identify the risk of subsequent rejection than Banff histologic scores, potentially leading to an earlier follow-up biopsy to detect subclinical rejection.

Currently, there are no validated diagnostic thresholds of the B-HOT gene score sets, limiting their interpretability.^17,23^ For this reason, we included the mean fold change in gene scores across histologic phenotypes in our analysis. Although expected test-to-test variation with the nCounter system assay has been reported, how variability translates to large gene sets (ie, there are 66 genes in the TCMR gene set) is unknown.^28^ Additional sources of test-to-test variability in the context of repeat measurements in the same patients over time include: (1) medication drug levels, doses, or adherence; (2) concomitant inflammatory events (ie, subclinical infections); (3) time from induction therapy or previous rejection treatments; and (4) biopsy sampling bias. Despite these limitations, in most cases but not all, the gene scores increased or decreased in relation to their histologic phenotype. The TCMR gene score had a mean fold change of 1.95 ± 0.87 when comparing no rejection to TCMR. The AMR, DSAST, and ENDAT gene scores had mean fold changes of 1.43 ± 0.24, 1.33 ± 0.22, and 1.17 ± 0.06, respectively, when comparing No Rejection to AMR (Figure 3).

The mean fold change in gene scores may be particularly relevant given the interindividual variability in gene scores identified in the baseline histologic no rejection state (Figure 7). A recent study by Foy et al^29^ demonstrated certain biomarkers to have patient-specific baselines that persist over decades and carry meaningful prognostic information. Whether this is true for molecular gene scores remains unknown. A recent large study has proposed a gene expression–based probabilistic scoring system.^22^ However, the added value of scores that are neither very high nor very low (eg, a biopsy with a 68% probability of TCMR) is unclear. An ongoing local study is currently investigating the variations in gene scores across a larger no rejection biopsy cohort. The mean fold change method for interpreting gene scores relies on the presence of a no rejection biopsy for comparison. The availability of such a biopsy may be limited to centers performing protocol biopsies. Diagnostic thresholds independent of the mean fold change would allow the B-HOT panel gene scores to be used more widely, but further study of individual baseline inflammation and gene expression is needed. Furthermore, although a large recent study has proposed a probabilistic scoring system, the added value of scores that are neither very high nor very low (ie, a biopsy with a 71% probability of TCMR) is unclear.^22^

Our study had the strength of including multiple biopsies performed over time with various histologic phenotypes, through both protocol and indication biopsies. Furthermore, pediatric biopsies were included and highly represented in our case series, with 46% of biopsies being from recipients younger than 20 y. This is unique, as the pediatric population is often underrepresented in the literature. Limitations of our study include that it is from a single-center and has a small population. Our case series consisted largely of White individuals, restricting generalizability. Furthermore, 6/8 study participants were pediatric at the time of transplant, with the youngest being 8.5 y at the time of transplant and 13.9 y at the time of the third biopsy. The greatest age difference was in a recipient who was 11.5 y at the time of transplant and 18 y at the time of the third biopsy. How aging and pubertal development might impact molecular phenotypes is unclear and could limit the generalizability of our findings. Although BK nephropathy was not present in any of the biopsies, we recognize that some patients may have transient BK viremia or infection with other viruses. Finally, as we were limited to biopsies performed as standard of care, there was significant time variation between biopsies, and whether time impacts such gene scores is unclear.

## CONCLUSIONS

Despite the significant interindividual variation in gene expression, the molecular phenotypes observed in renal transplant biopsies exhibit dynamic changes in response to the histologic phenotype over time. There is potential to use the B-HOT panel molecular gene scores to facilitate the diagnosis of rejection states in complement to histology. However, further study of gene expression at various time points, including baseline, after protocol biopsies, and following clinical events, will enhance the utility of this promising technique within the transplant community.

## Supplementary Material


