# Supplementary material for: The Evolution of Molecular Phenotypes Through Time in Kidney Transplant Recipients
Source: Transplant Direct. 2026 Mar 23;12(4):e1919. doi: 10.1097/TXD.0000000000001919 (PMC13012213; doi:10.1097/TXD.0000000000001919)
Supplement: Supplementary file 1 [file txd-12-e1919-s001.pdf]

Supplemental Table 1. Molecular gene score references, clinical relevance, and genes comprising the score.

	Gene score reference	Reported clinical relevance
**TCMR score**	Smith RN, Rosales IA, Tomaszewski KT, et al. Utility of Banff Human Organ Transplant Gene Panel in Human Kidney Transplant Biopsies. *Transplantation*. 2023;107(5):1188-1199. doi:10.1097/tp.0000000000004389	Distinguishes histologic TCMR and Mixed Rejection from ABMR alone[1]
**ABMR score**	Mengel M, Loupy A, Haas M, et al. Banff 2019 Meeting Report: Molecular diagnostics in solid organ transplantation–Consensus for the Banff Human Organ Transplant (B-HOT) gene panel and open source multicenter validation. *Am J Transplant*. 2020;20(9):2305-2317. doi:10.1111/ajt.16059	Distinguishes histologic ABMR and Mixed Rejection from TCMR alone.[1]
**DSAST score**		Distinguishes histologic ABMR and Mixed Rejection from TCMR alone.
**ENDAT score**		Associated with ABMR, HLA class II antibodies, and graft loss.

Supplemental Table 2. Individual genes comprising molecular gene scores.

TCMR (n=66)		ABMR (n=32)	DSAST (n=19)	ENDAT (n=33)
ADAMDEC1	IFNG	ACKR1	ACKR1	ACKR1
AIM2	IL18BP	CAV1	CDH13	ADORA2A
ANKRD22	IL23A	CD34	CDH5	BMP6
AOAH	IL2RA	CDH13	COL13A1	CARD16
BATF	IL2RB	CDH5	CX3CR1	CASP1
BTLA	KLRD1	CX3CR1	FGFBP2	CAV1
C1QA	LAG3	CXCL11	GNG11	CD34
C1QB	LAIR1	FGFBP2	GNLY	CDH5
CCL5	LAP3	GATA3	ICAM2	CETP
CCR5	LCK	GNLY	KLRF1	COL4A1
CD2	LILRB4	IFNG	MALL	ICAM2
CD27	MS4A6A	KLF4	MYBL1	KDR
CD28	MYB	MALL	PLA1A	KLF4
CD3D	PDCD1LG2	MYBL1	PLAT	MCAM
CD3G	PSMB10	PALMD	ROBO4	MEOX1
CD6	PSMB9	PECAM1	SH2D1B	NOD1
CD72	PSME2	PLA1A	SOX7	NOS3
CD84	PSTPIP1	PLAT	TEK	NOX4
CD86	PTPN7	PSMB10	TM4SF1	NR4A1
CD8A	SH2D1A	RHOJ		PALMD
CD8B	SIRPG	ROBO4		PDGFRB
CD96	SLA	RPS6		PECAM1
CTLA4	SLAMF6	RPS6KB1		RASIP1
CXCL13	SLAMF8	SELE		RGS5
CXCR6	SP140	SH2D1B		RHOJ
DUSP2	ST8SIA4	SOX7		S1PR1
EOMES	STAT1	TBX21		SELE
FASLG	TAP1	TEK		SELP
FCGR1A	TIGIT	THBD		SERPINE1
GZMK	TLR8	TNF		TEK
HLA-DMB	TNFRSF9	TRIB1		THBS1
HLA-F	TOX2	VWF		VCAM1
ICOS	ZAP70			VWF

Supplemental Table 3. Donor-specific antibody (DSA) characteristics among recipients (n=8) with serial biopsies.

Subsets	n	MFI of the highest bead (mean)	ABMR gene scores* (mean)	DSA gene score* (mean)
**DSA Class**				
Class I alone	1	2194	41	35
Class II alone	2	4136	38	31
Class I and class II	5	7258	37	29
**DSA class II subtype**				
DP alone	0	-	-	-
DQ, no DR, ± Class I	3	2706	39	31
DR, no DQ, ± Class I	1	2216	28	20
DR and DQ, ± Class I	3	8287	39	35
**DSA specificity**				
A1, A68	1	2194	41	35
A34, DR15, DR51, DQ5	1	2174	40	31
B62, DR17, DQ2, DR53	1	12341	37	32
DQ2, DR17	1	10347	40	30
DQ5	1	1779	39	29
DQ6	1	2404	34	29
DQ7	1	3936	45	35
DR13, DRB3*03:01	1	2216	29	20

* All subset comparisons were insignificant ($p>0.05$)
